# Pretreatments and Particle Size on the Glycemic Index and Rheological and Functional Food Properties of Bean Flours

**DOI:** 10.1155/2024/6336837

**Published:** 2024-05-20

**Authors:** Adriana Mejía-Terán, Carla María Blanco-Lizarazo, Jairo Eduardo Leiva Mateus, Indira Sotelo-Díaz, Darío Mejía Terán, Enrique Geffroy

**Affiliations:** ^1^Doctorado en Ciencias Naturales para el Desarrollo (DOCINADE), Instituto Tecnológico de Costa Rica, Universidad Nacional, Universidad Estatal a Distancia, San Jose, Costa Rica; ^2^Grupo Interinstitucional de Investigación en Ciencias Agropecuarias, Forestales y Agroindustriales del Trópico, Universidad Nacional Abierta y a Distancia (UNAD), Bogotá, Colombia; ^3^Centro de investigación y desarrollo cárnico CI+D, Industria de Alimentos Zenú, Medellín, Colombia; ^4^Instituto de Investigaciones en Materiales (IIM), Universidad Nacional Autónoma de México (UNAM), Ciudad de México, CDMX 4510, Mexico; ^5^Grupo de Alimentación, Gestión de Procesos y Servicio, Universidad de La Sabana, Chía, Colombia; ^6^Grupo de Estudios Ambientales Aplicados, Universidad Nacional Abierta y a Distancia (UNAD), Bogotá, Colombia

## Abstract

The beans' protein and slow-digesting carbohydrate content make it an appealing choice for healthy food development. However, its properties are influenced by the flour extraction processes. This study is aimed at evaluating the effect of particle size and three pretreatments—drying (D), soaking + cooking + dehydrating 3 h (SCD3), and soaking + cooking + dehydrating 24 h (SCD24)—on the estimated glycemic index (eGI) compared with raw bean flour (R). The methodology covered water absorption (WAI), water solubility (WSI), amylose content, starch digestibility, eGI, phenolic quantification, and rheology. The results showed that WAI correlated negatively with WSI and amylose, varying among pretreatments and sizes. WAI increased as D < SCD24 < SCD3 < R. Glucose release (HI) differed between fine (125 *μ*m) and coarse fractions (242 *μ*m), with SCD24 and R showing the lowest eGI (22.8–24.2). SCD3 had the highest flavonoid concentration, while R and D had more quercetin-3-glucoside. SCD24 displayed higher elastic/viscous moduli than R. Bean flours from all treatments had low GI and contained bioactive polyphenols (catechin, epicatechin, ferulic acid, quercetin). The optimal treatment was SCD24, particularly in the coarse fraction, showing potential for functional food development and novel applications such as precision nutrition.

## 1. Introduction

Current food trends reveal a prominence of low-calorie, low-glycemic, and gluten-free foods linked to functional benefits in health and precision nutrition [1]. Among legumes, beans are an attractive option for food design due to their high protein and slow-digesting, nondigestible carbohydrate content, which ferments in the large intestine as resistant starch and nondigestible oligosaccharides [2]. They also contain phenolic compounds such as flavonoids and phenolic acids that may have *α*-amylase, *α*-glucosidase, and lipase inhibitory activity, as well as antioxidant properties, potentially beneficial for diabetes control, obesity prevention, and tailored nutrition prescription as a promising approach in nutrition precision practices [3, 4]. Thus, the compounds present in beans potentially have health benefits, the effects of which are directly proportional to their intake [5]. Therefore, converting legumes into flour has been identified for technological use and as food additives, thereby increasing their application in processed foods [6, 7].

However, legumes contain antinutritional factors such as protein compounds (lectins, protease inhibitors, or antifungal peptides) and nonprotein compounds (phytic acid, alkaloids, and specific phenolic compounds), which reduce the absorption and decrease the digestibility and the bioavailability of some nutrients, which could be toxic and cause physiological discomfort when raw seeds or flours are consumed; additionally, they might impart bitter flavors and nonpalatable effects to foods [8]. Thus, to inactivate or reduce their antinutritional effects, whole beans are pretreated, generally using thermal processes, before milling [9]. It has been suggested that combined pretreatments that include at least three processing methods, such as soaking, pressure cooking, and dehydration, effectively reduce the inhibitory activity of nonnutritional or toxic compounds [10]. However, pretreatments can also modify the digestibility of legume flours and promote molecular physical interactions between starch and proteins, which correlate with starch nutritional fractions (SDS-RS), hydrolysis rate, and glycemic index [11].

Accordingly, it has been reported that the physicochemical and functional characteristics of flours are governed by differences relating to variety, heat treatments, and milling methods [9, 12, 13]. Factors such as flour particle diameter have been shown to influence the nutritional composition, water-holding capacity, and starch digestibility of the fractions [14]. Guo et al. [15] found that raw wheat flours presented lower digestion rates in the larger particle size fractions (250–500 *μ*m); furthermore, the resistant starch (RS) content of coarse flours was higher than that of fine flours. These results are attributed to the more disrupted structure and greater surface area of particles of fine flours, which increases their susceptibility to amylolysis. Other studies show high variability in the digestibility rate of legume flours depending on process variables that disrupt cell structure, which could affect the modification of starch hydrolysis and the glycemic index. These changes induce modifications in the biopolymers and affect the food matrix's nutritional, technological, and functional properties [16, 17]. However, the literature on the impact of the synergy of pretreatments and particle size on the technological characteristics of legume flours and the postprandial glycemic index is scarce.

In this study, a new Colombian bean was investigated: the UNAD bean variety, *Dosq-Zandú* (*breeder certificate A182474*), was selected due to its adaptability to temperate and cold climates, alongside its remarkable disease tolerance. Developed through reciprocal crossing, this bush variety reduces environmental impacts by eliminating the need for support structures and reducing the use of agrochemicals. Its morphoagronomic characteristics, such as large kernels and attractive color, are highly valued by consumers, and its high yield holds the promise of economic benefits. Additionally, its chemical composition contributes to a beneficial nutritional profile. This selection was made based on its multiple agronomic, economic, and environmental advantages.

The pretreatments and their process variables were selected to improve the nutritional and technological factors of the bean flour. The soaking treatment was based on the results by Naiker et al. [7], aimed at rehydrating the beans, activating endogenous phytase, and reducing cooking times. Additionally, the pressure cooking process was applied to reduce antinutritional factors, as cooking under pressure could allow for heating rates, thereby reducing the nutrient loss. Moreover, this pretreatment was associated with an increase in the resistance fraction of enzymatic hydrolysis, as gelatinized starch polymers could retrograde to a less soluble form. The drying process was chosen to increase the content of RS fractions and improve the bioavailability of phenolic compounds [7, 18–20].

Consequently, this research is aimed at evaluating the glycemic index, technological properties, composition and concentration of phenolic compounds, and the rheological properties in bean flours with different particle sizes and three pretreatments (soaking, cooking, and dehydration) compared with raw bean flours.

## 2. Materials and Methods

### 2.1. Materials

The UNAD-DOS ZANDU bean variety, provided by the Agriculture and Biotechnology Research Center and the bean improvement program at Universidad Nacional Abierta y a Distancia, UNAD, Dos Quebradas, Risaralda, Colombia, was studied (4°50′19^″^ N 75°40′13^″^ O). The proximate chemical composition of UNAD-DOS ZANDU beans was 37.67 ± 0.9% native starch, 4.61% total sugars, 22.07% ± 0.21 crude protein, 0.50 ± 0.01% fat, 5.13 ± 0.21% natural fiber, 3.39 ± 0.09% ash, and 14.83 ± 0.12% moisture. All analytical tests were performed randomly on three independent bean lots.

### 2.2. Experiment Design

This study was conducted in three phases. The first phase consisted of a completely randomized design with two factors corresponding to pretreatment and particle size; each analysis was performed in triplicate from three independent lots of beans.

1. Phase 1: the WAI and WSI of various bean flours were assessed using a completely randomized design (4 × 5). These factors corresponded to different pretreatment methods for obtaining bean flour and particle diameters. The pretreatment methods comprised four levels: drying (D), soaking + cooking + dehydration 3 h (SCD3), soaking + cooking + dehydration 24 h (SCD24), and raw flour (R) as the control. Particle diameters were evaluated at five levels: 500 *μ*m, 425 *μ*m, 300 *μ*m, 212 *μ*m, and 150 *μ*m.
2. Phase 2: the dependent variables were the digestibility ate, eGI, starch fraction ratios, amylose/amylopectin percentages, and phenolic compounds quantified. The particle diameters of each flour were grouped according to the results of WAI and WSI. Consequently, bean flours (SCD3 and SCD24) were classified into whole, coarse, and fine fractions, grouping them by means of an orthogonal contrast test and evaluating significant differences between the particle sizes studied. Control R and pretreatment D flour did not present statistically significant differences among their fractions. Thus, the whole flour was evaluated with no fractionation.
3. The average particle diameter for whole, coarse, and fine fractions for SCD3 was 163.8 *μ*m, 192.7 *μ*m, and 125 *μ*m, respectively; and for SCD24, it was 161 *μ*m, 242 *μ*m, and 125 *μ*m, respectively. Whole R and D bean flours had an average particle diameter of 179.1 *μ*m and 189.4 *μ*m, respectively.
4. Phase 3: drawing from the results of the phase 1 and 2 results, the rheological behavior, flow measurement, strain sweep, frequency sweep test, and temperature ramp were conducted on the SCD24 coarse fraction (SCD24_C) and whole bean flours (R) due to their demonstrated lower eGI.

### 2.3. Pretreatments for Flour Production

For control R, raw beans were ground; for pretreatment D, beans were dried at 120°C for 30 min in a Heratherm forced convection tray oven (Thermo Scientific, Germany). For pretreatments SCD3 and SCD24, beans were soaked in water (1 : 3 w/v) at room temperature for 12 h and then cooked in hot water in a pressure cooker (Imusa, Colombia) for 30 min. Then, they were washed with cold water at 4°C for 2 min. SCD3 beans were dried at 120°C for 3 h, while SCD24 beans were dried at 75°C for 24 h. Both pretreatments were performed in a Heratherm forced convection tray oven (Thermo Scientific, Germany).

During soaking, the presence of off-flavors related to fermentation was verified. To prevent this process, the microbiological quality of the beans was assessed, and good practices for production, storage, and handling were implemented. Additionally, potable water was used.

After applying the pretreatments to the beans, dry grinding was carried out for 2 min in a 5.29 oz electric grain pulverizer mill (Cgoldenwall, China). The flours were fractionated sequentially on the series of five ASTM (U.S. Standard) sieves for 6 min in a Vibratory Sieve Shaker, Analysette 3 Spartan (Fritsch, Germany). All flours and fractions were vacuum-packed in low-density polyethylene plastic bags and stored at 4°C until analysis.

### 2.4. Technological Characterization of Flours

#### 2.4.1. Water Absorption Index (WAI) and Water Solubility Index (WSI)

WAI and WSI were determined according to the method proposed by [21] with minor modifications. A 1 g sample of each flour fraction was dispersed in 10 mL of distilled water at 30°C and stirred for 30 min at 400 rpm on a Velp Arec magnetic stirrer with heating (VELP Scientifica, Italy). Water was added to the dispersions to bring them up to 13 mL, and these were then centrifuged at 1700 g for 10 min in a Power Spin DX centrifuge (UNICO, New Jersey, USA). The supernatant was decanted to determine the total dissolved soluble solids, and the sediment was weighed according to the methodology proposed by Igual et al. [22]. The WAI corresponded to the weight of sediment following the removal of the supernatant per unit of weight of the original sample. The WSI corresponded to the weight of dissolved solids in the supernatant measured in a Brix digital refractometer in the 0–85% range (Milwaukee Instruments, Wisconsin, USA) and expressed as a percentage of the sample's original weight.

### 2.5. Analysis of Functional Properties

#### 2.5.1. Amylose/Amylopectin Content

The amylose content of the flours was determined following the protocols by Megazyme® using the amylose kit K-AMYL 06/18 (Megazyme, Wicklow, Ireland). Lipids were removed by precipitating the starch in ethanol and recovering the precipitated starch. Lectin concanavalin A was added for the specific synthesis of amylopectin complexes, which were removed by centrifugation at 3000 g for 20 min in a Power Spin™ DX centrifuge (UNICO, New Jersey, USA). Subsequently, the enzymatic hydrolysis of amylose to D-glucose was performed and analyzed using GOPOD reagent by colorimetric quantification with the D-Glucose Assay kit (Megazyme International, Wicklow, Ireland).

#### 2.5.2. Quantitative Analysis of Phenolic Compounds

Phenolic extracts were obtained in a methanol mixture: 0.2% water in formic acid (1 : 1), vortexing (5 min), and sonication (5 min); then, these were quantitatively analyzed using an ultrahigh performance liquid chromatography (UHPLC), Dionex Ultimate 3000 (Thermo Scientific, Sunnyvale, CA, USA). Mass spectra were acquired in the mass range m/z 60–900. Compound identification was performed using full-scan acquisition mode and ion extraction (EIC) corresponding to the [M + H]^+^ of the compounds of interest, measuring mass with accuracy and precision of Δppm < 1 and using a standard solution-mix of the phenolic compounds.

#### 2.5.3. *In Vitro* Digestion of Starch and Calculation of Starch Fractions

The *in vitro* starch digestion was determined according to the protocol proposed by Englyst et al. [23] with some modifications. A 2-phase digestion procedure involving a gastric and a pancreatic phase was performed. In the simulated gastric digestion, the flour samples were incubated in a 5 mL solution of 0.05 M HCl, pH 1.5, with 7 mg pepsin for 60 min at 37°C. To simulate the pancreatic phase, the pH was fitted to 5.2 with 0.5 M sodium acetate buffer (3.5 mL) with the addition of pancreatic amylase (10 mg), amyloglucosidase (0.06 mL), and invertase (0.086 mg); the enzymes used were from Sigma-Aldrich (St. Louis, USA). The glucose released from the sample was determined colorimetrically with the glucose oxidase/peroxidase (GOPOD) reagent using the D-Glucose Assay Kit (Megazyme International, Wicklow, Ireland) at 0, 20, 60, 120, and 180 min. The contents of rapidly digested starch (RDS, hydrolyzed after 20 min), slowly digested starch (SDS, hydrolyzed after 120 min), and resistant starch (RS, not hydrolyzed after 120 min) were calculated. The corn starch (high amylose, 68%) was used as standard (Megazyme International, Wicklow, Ireland).

#### 2.5.4. Estimated Glycemic Index (eGI)

The eGI was determined by the *in vitro* method proposed by Granfeldt et al. [24] based on each sample's hydrolysis index (HI). The HI was calculated as the ratio between the area under the hydrolysis curve (0–180 min) of the sample and the area under the curve of high amylose (68%) corn starch standard (Megazyme International, Wicklow, Ireland).

#### 2.5.5. Rheological Properties

The doughs were prepared based on preliminary tests and the flours' water absorption index, using deionized water at 25°C in a 1 : 1.3 flour-to-water ratio. The samples were reposed for 30 min before analysis.

The rheological properties of the samples R and SCD24_C were determined using an ARES-G2 controlled-stress rheometer (TA Instruments, New Castle, DE, USA) equipped with parallel plates (25 mm diameter, 2 mm spacing). Approximately 982 mm^3^ of the sample was loaded into the plates for measurement. The top plate was positioned over the sample slowly (0.001 mm s^−1^) to minimize extra stresses due to compression. Subsequently, the excess sample was wiped off, and the sample was left at rest for five minutes before measurements were started. This procedure was repeated for each test performed. The rheological behavior was studied through two types of tests: flow (viscosity vs. shear rate) and oscillatory (frequency, strain sweeps, and temperature ramp).

A shear rate of 0.1 to 10 s^−1^ was applied for the flow measurement, and the data obtained were fitted to the power law model proposed by Ostwald–de Waele; the strain sweep test was evaluated with a strain oscillation between 0.01 and 10%; frequency sweeps were performed over an angular frequency range of 0.1 to 100 rad/s, with 1% strain set within the linear viscoelastic region at 25°C. Temperature ramp tests on the doughs were carried out in a temperature range from 25°C to 90°C then cooled to 25°C at a rate of 2°C min^−1^, constant frequency (*ω*) 6.28 rad/s (1 Hz), and 0.05% strain. The storage modulus (*G*′), the loss modulus (*G*^″^), and the value of the loss tangent (tan(*δ*) = *G*^″^/*G*′) were obtained from these rheological measurements. Texture maps and material classification were built according to Schreuders et al. [25].

### 2.6. Statistical Analysis

Phase one of the statistical analysis was performed using a two-way analysis of variance, Duncan's multiple comparison test, and orthogonal contrasts (*α* = 0.05) to analyze differences between means on WAI and WSI and their interactions. A one-way analysis of variance and Tukey's multiple comparison test (*α* = 0.05) were performed for the second phase. A Pearson and Spearman correlation analysis was performed to establish possible linear and monotonic linear relationships between the variables evaluated. SAS ODA statistical software was used for all statistical procedures (SAS Institute, Inc., Cary, NC).

## 3. Results and Discussion

### 3.1. WAI and WSI

**Figure 1:**
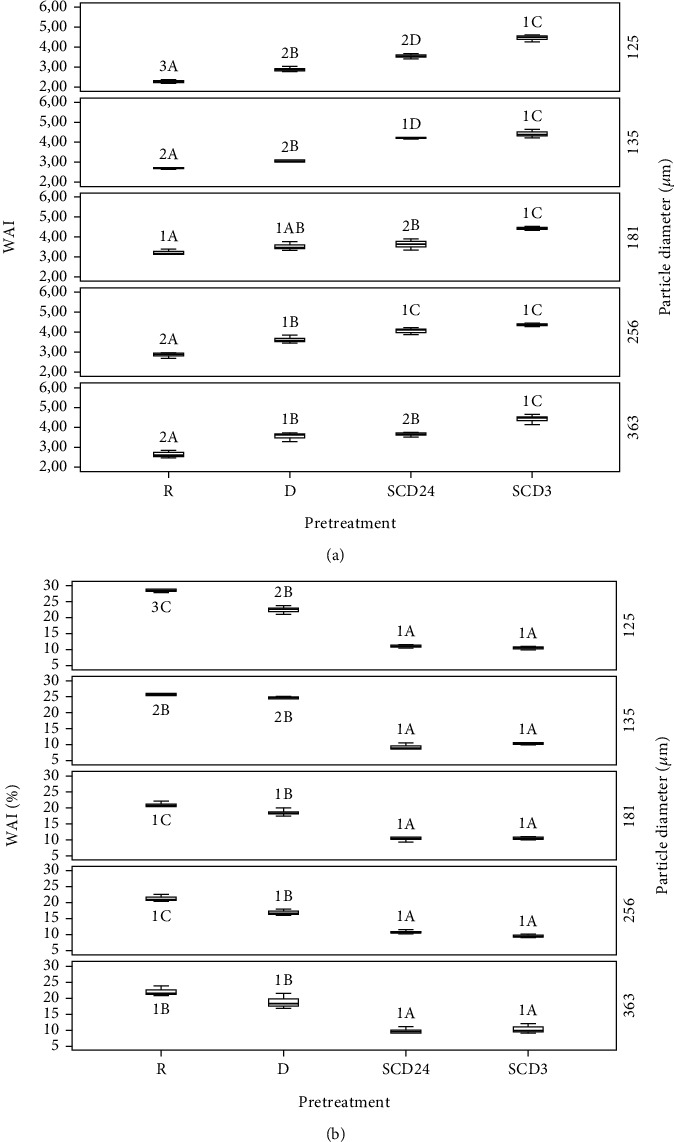
(a) Water absorption index (WAI) and (b) water solubility index (WSI) for bean flours, by different pretreatments and particle size: raw (R), drying (D), soaking + cooking + dehydration per 24 h (SCD24), and soaking + cooking + dehydration per 3 h (SCD3). (A–D) Different letters show statistically significant differences between pretreatments (*P* > 0.05) by Duncan. (1, 2) Different numbers show statistically significant differences between particle diameters (*P* > 0.05) by Duncan.

Figure 1 shows the WAI and WSI values for each pretreatment by particle size of the bean flours and the control (R). For the WAI values of the bean flours, there are highly significant differences between pretreatments and particle sizes (*p* < 0.0001) (Figure 1(a)), as well as significant interactions between both factors (*p* < 0.05). Thus, WAI significantly increases with the pretreatment in ascending order for D, SCD24, and SCD3, as compared to R for all the particle diameters evaluated (*p* < 0.05). The 125 *μ*m fraction of control R presented the lowest WAI (2.27) with significant differences (*p* > 0.05) from the flours with the pretreatments. However, there were no significant differences (*p* > 0.05) between particle sizes for SCD3. There were no significant differences (*p* > 0.05) between fractions 256 *μ*m and 363 *μ*m in pretreatment (D) and control (R).

Likewise, a negative correlation between WSI and WAI was observed in bean flour (*r* = −0.83; *p* = 0.0001). Thus, WAI decreases when WSI increases as the particle sizes of the fractions decrease.

The decreasing WAI in the smaller particle size of the fractions coincides with the results found by Feng et al. [26], who suggest that reducing particle size through grinding increases water absorption due to the destruction of structural integrity, leading to the breaking of hydrogen bonds and exposing higher levels of hydrophilic groups. Additionally, bean flours with smaller particle diameters exhibit greater surface area, thereby increasing the potential for surface water absorption, which limits starch gelatinization and reduces WAI. However, the increased surface area also enhances starch exposure, allowing for the formation of dextrins and simple carbohydrates, thereby increasing WSI [27].

The average WAI value for R was consistent with Kenar et al.'s [28] reported values of 2.74 in raw white bean meal, and Wani et al. [29] report values between 2.6 and 2.7 in raw kidney bean meal. WAI values increased in bean flours pretreated at high temperatures compared to raw flour. This behavior was concordant with that found by Naiker et al. [7] for hyacinth bean flours, where the water absorption capacity increased significantly from 0.72 to 2.66 in raw flour due to the effect of soaking and cooking at high pressure and dehydration treatments. This phenomenon could be attributed to protein denaturation and unfolding processes that expose peptide bonds, which would provide greater accessibility to the polar groups of amino acids and an increase in the surface-to-mass ratio linked to an increase in noncovalent hydrogen bonds between polypeptide chains and water molecules [13, 28]. Furthermore, thermal pretreatments of SCD could have increased starch dextrinization and amylopectin decomposition. These phenomena could lead to starch gelatinization and increased interaction with water [20].

The average WSI values for bean flours fluctuated between 9.66 and 28.47% (Figure 1(b)). This technological variable presented highly significant differences between pretreatments and particle diameters (*p* < 0.0001). The interactions between both factors were also highly significant (*p* < 0.0001). However, the R bean control presented the highest magnitudes for this index. It was also observed that there were no statistically significant differences (*p* > 0.05) between the particle sizes evaluated for SCD3 and SCD24.

The control flour R presented the highest average WSI value of 23.04%. This value is similar to that for raw white bean flours, as reported by Kenar et al. [28], who reported higher WSI (17.6%) compared to pinto bean flours processed by jet-cooking at 138°C and drum-drying. The WSI value for R is higher than that of processed flours because raw flours are probably heterogeneous and contain lower-molecular-weight soluble components, such as native starch. In addition, raw flours may contain native proteins that, being more soluble, leach more easily when they have not been heat-treated. In contrast, pretreated flours may contain a mixture of polymers and low molecular weight solutes that could be trapped in the matrix, including amylose, amylopectin, and denatured proteins due to heat. This can hinder the solubility of their compounds. The phenomenon of trapped matrix could be associated with protein denaturation and starch gelatinization, where thermochemical transformations increase hydrogen bonds, hydrophilic interactions, and ionic forces between compounds [30].

### 3.2. Determining Amylose Content

Regarding the amylose content (Table 1), the highest values (54.97–67.93%) found were for control R and bean flour from pretreatments D, SCD24_Whole, and SCD24_Coarse, with no significant differences among them (*p* > 0.05). Amylose content had a negative correlation with WAI (*r* = −0.57; *p* = 0.0062) and a positive correlation with WSI (*r* = 0.52; *p* = 0.0072).

On the other hand, the amylose contents in the bean flours were found to be in the range of the values for Carioca bean starch extracts analyzed by Los et al. [31], i.e., between 40.06% and 42.60%. Thus, amylose content is a relevant parameter for food design because it affects technological properties such as water solubility index (WSI) and water absorption index (WAI), as shown with the flours evaluated in our study. Accordingly, R, D, and whole SCD24 bean flours, which have higher amylose contents, would have a higher shear strength and be more susceptible to starch retrogradation and minor starch swelling [32].

### 3.3. Phenolic Compounds

Table 2 shows the concentration and type of phenolic compounds identified in the bean flour. Here, the fine and coarse fractions of SCD3 had the highest concentration of flavonoids, with significant differences compared to the other pretreatments in the catechin and epicatechin concentrations (*p* < 0.05). Pretreatment D had the highest concentration of quercetin-3-glucoside, without significant differences with control R but with significant differences with the other pretreatments (*p* < 0.05). Regarding ferulic acid concentrations, these decreased statistically (*p* < 0.05) for SCD24 compared to the other treatments. Additionally, pelargonidin-3-glucoside was only found in control R and pretreatment D at 14.95 mg kg^−1^ and 25 mg kg^−1^, respectively. There were no significant differences (*p* > 0.05) between the concentration of phenolic compounds in the fine and coarse fractions in SCD3 and SCD24.

Regarding the bioactive potential of bean flour, samples from all pretreatments show catechin, epicatechin, and quercetin contents, which have been reported as the main phenolic compounds in legume grains [19]. The SDC3 pretreatment presented the highest concentrations of flavonoids, possibly derived from the rupture of the cell walls and compartments, improving their bioavailability, which may occur in the drying phase at 120°C for three hours [33, 34].

During soaking, phenolic compound may be reduced due to their migration into the water [35]. However, the SDC3 treatment exhibited the highest concentration of phenolic compounds, which can be attributed to the cooking effect and short drying time. Processing at high temperatures may release phenolic compounds accumulated in the vacuoles of the beans, and high temperatures can also deactivate oxidative and hydrolytic enzymes that could otherwise degrade phenolic compounds [18, 36]. For the SCD24 treatment, the reduction in phenolic compound concentration in both fractions may be related to their degradation during prolonged drying. These findings are consistent with reports by Ramírez-Jiménez et al. [19] on black beans, where flavonoid concentrations (such as quercetin and rutin) increased after cooking (boiled at 94°C for 2.5 h) and drying (oven-dried at 60°C for 12 h). However, reports on the influence of thermal treatments on phenolic compounds are contradictory [37].

Ferulic acid was identified in all pretreatments at a higher concentration of 4.16 *μ*g g^−1^, as found by Ramírez-Jiménez et al. [19] for black beans. The concentration of this phenolic acid may be related to decreased eGI for bean flours due to a mixed-type inhibitory effect against *α*-amylase and a noncompetitive type of inhibition mechanism against *α*-glucosidase with interaction forces that may be hydrogen bonding. This phenomenon has been reported to be linked to bean flour's potential for regulating the postprandial glycemic level [38, 39]; in this way, the pulse powders could bring nutritional and functional advantages to mainstream food products [40].

The phenolic compounds present in beans have been shown to exhibit inhibitory activity against *α*-amylase, contributing to the delay in starch digestion and affecting the glycemic index [3]. However, it is important to consider the impact of the digestion on phenolic compounds in terms of bioaccessibility and bioavailability [41, 42].

### 3.4. Determining Starch Fractions

Table 1 shows the percentages of RDS, SDS, and RS fractions and amylose content. Considering the RDS fraction, the highest values (13.91–16.35%) were for the fine particles (125 *μ*m) of SDC3, SDC24, and whole SDC24 (161 *μ*m) with no significant differences between them (*p* > 0.05).

Likewise, the RS in fractionated flours SDC24 and SDC3 increased as a function of particle diameter with statistically significant differences between the fractions (*p* > 0.05). The highest RS values (30.55–28.57%) were for coarse SDC24 and whole raw flour. Regarding functional characteristics, the percentage of RS in bean meal was consistent with the data found by Chung et al. [12] and Hooper et al. [43], where the fractions of RS ranged from 32% to 36% for bean flours.

### 3.5. *In Vitro* Starch Digestibility and Estimated Glycemic Index

**Figure 2:**
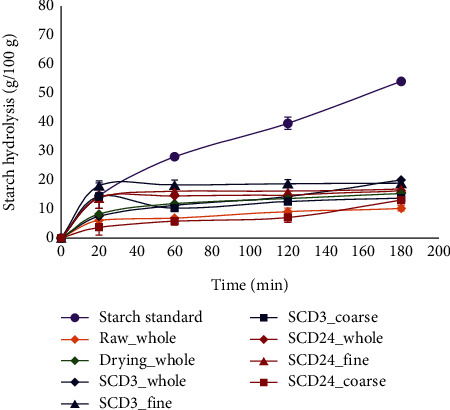
*In vitro* starch digestion curves of the bean flours, by different pretreatments.

Figure 2 shows the *in vitro* starch digestibility curves of bean flours with different pretreatments. The release of glucose quantified in HI in the digestion time studied showed statistically significant differences between the fine and coarse fractions (*p* < 0.05). The fine fractions of SDC3 and SCD24 presented the highest starch hydrolysis (16.49-20.05%). This behavior could be explained by the hydrolyzation of the digestible starch over the first 20 min (between 80.68 and 95.41%), followed by a stable behavior. The coarse fractions of SCD24 showed lower starch digestion rates after 180 minutes (10.21–13.11%) with no significant differences with the control R but with statistically significant differences with the other pretreatments (*p* < 0.05).

**Figure 3:**
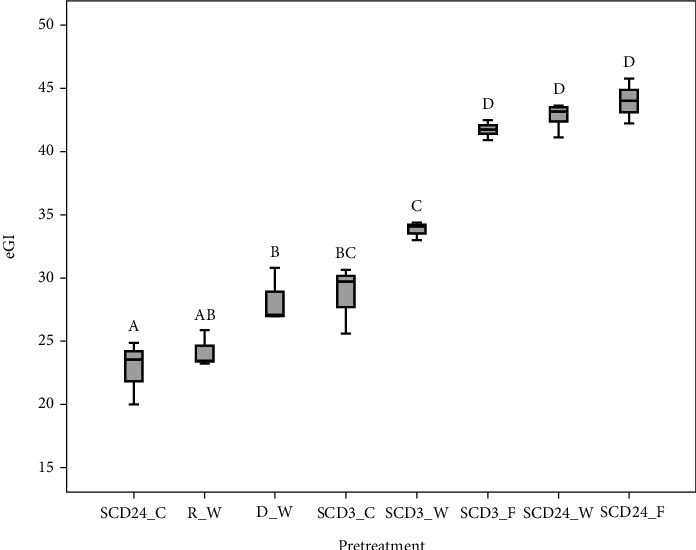
Estimated glycemic index (eGI) for bean flours, by pretreatments and particle size. Pretreatments: raw (R), drying (D), soaking + cooking + dehydration 3 h (SCD3), and soaking + cooking + dehydration 24 h (SCD24). Particle size: whole (W), fine (F), and coarse (C). (A–D) Different letters show statistically significant differences between pretreatments and particle size. *P* < 0.05 by Tukey.

At 180 minutes, all pretreatments were digested at a lower starch hydrolysis (10.21–20.05%) than the corn starch standard (53%), with statistically significant differences (*p* < 0.05). The average eGI of the bean flours ranged between 22.82 and 44.02 (Figure 3). The pretreatment SCD24_Coarse presented the lowest eGI corresponding to 22.82 without significant statistical differences (*p* > 0.05) concerning the control R with an eGI of 24.16. However, it showed statistically significant differences (*p* < 0.05) compared to the other pretreatments. For bean flour, RS content demonstrated a positive correlation with WSI (*r* = 0.58, *p* = 0.0028) and a positive correlation with amylose content (*r* = 0.53, *p* = 0.0017). Regarding eGI, negative correlations of this parameter with RS were found (*r* = −0.80, *p* < .0001) while a positive correlation with RSD was shown (*r* = 0.89, *p* < 0.0001). However, pretreatment and particle diameter influenced the digestion rate and, consequently, the eGI in bean flours. This behavior could be attributed to the fact that heat treatment during this pretreatment may produce a change in bean structure due to denaturation and aggregation of cell membranes. On the other hand, the thermal treatment influences the structure and texture of cells, causing the loss of firmness and aiding in amylase access [6, 7]. Also, the thermal treatment could increase the granules' degree of gelatinization and swelling which, added to the changes in the crystalline structure, increases the accessibility to the starch molecules, causing an increase in the digestibility rate with respect to that raw flour.

The protein matrix could encapsulate the starch granule, preventing starch swelling and gelatinization and limiting the accessibility to attack by amylolytic digestive enzymes [44–46].

The results for the coarse flours with particle sizes larger than 192.7 *μ*m showed lower digestibility rates compared to the fine fractions, which could be attributed to the decrease in the ratio between the surface area and the volume, reducing the surface on which starch granule hydrolysis by the amylolytic enzymes takes place [47]. The finer fractions would have smaller, porous starch granules with lower crystallinity and a thin layer structure, causing a different mode of *α*-amylase and amyloglucosidase activity, according to the granule size [48].

### 3.6. Rheological Properties

#### 3.6.1. Flow Measurement

**Figure 4:**
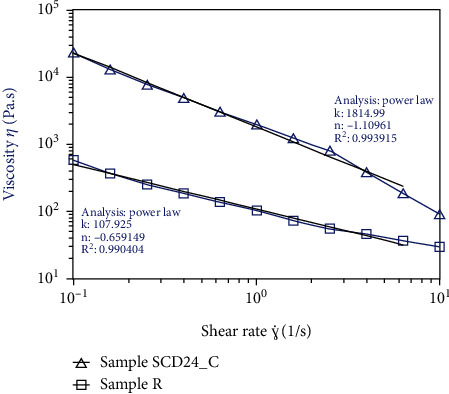
Flow behavior of bean doughs SCD24_C and R and influence of shear rate on the viscosity curve at 25°C. The data were fitted to the power law model, where k means the consistency coefficient and n means the flow behavior index.

Figure 4 shows the flow curve for two doughs (R and SCD24_C). The behavior was similar for the two samples, with the slope monotonically decreasing. The increasing shear rate (*γ*) in the two samples led to decreased viscosity (*η*) in the range of 10s^−1^ to 10, revealing that the samples had a shear-thinning behavior, characteristic of many non-Newtonian foods. The flow model used in this study was the power law, which is used to describe pseudoplastic behavior and showed a fitting of flow (*R* > 99%) for the two samples. The R and SCD24_C doughs showed *n* values <1 (-0.65, -1.109), confirming the shear-thinning behavior of bean flours.

Regarding the flow behavior of SCD24_C and R, the samples experienced deformation as the shear rate increased, which was consistent with the results found by Lin and Fernández-Fraguas [13], who reported a similar shearing result in dispersions of common beans, showing characteristics of a pseudoplastic material. This property could be attributed to the fact that the molecular structure of the material began to break down, which reduced internal resistance in the form of friction and resistance to flow.

The SCD24_C sample showed the highest viscosity values compared to the crude R dough, which could be because the starch was precooked and has greater swelling, greater water-holding capacity, and resistance to the dispersion to flow; it also can present a high cross-linked structure that must be broken, indicating that the pretreatment had a significant effect on the structure of the starch and the interaction of its components; consequently, it contributes to the maximum viscosity of the dough [49, 50].

#### 3.6.2. Strain Sweep

**Figure 5:**
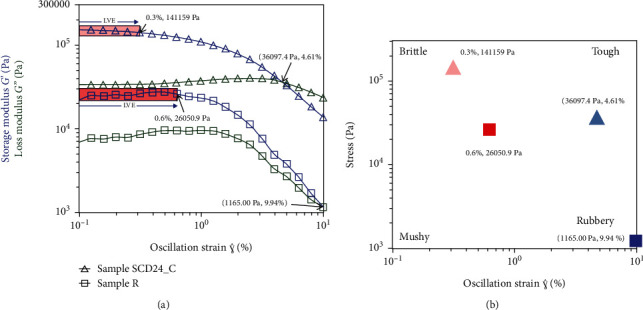
Dynamic strain sweep for R and SCD24_C. (a) Storage modulus (*G*′ (blue)) and loss modulus (*G*^″^ (green)) versus oscillation strain to define the end of the linear viscoelastic regime and the crossover point (*G*′ = *G*^″^). (b) Texture map and the classification of materials in four quadrants using the stress rates with the oscillation strain at the limit of the linear viscoelastic (LVE: R (red square), SCD24_C (pink triangle)) region and the crossover point (*G*′ = *G*^″^: R (light blue triangle), SCD24_C (blue square)).

Figure 5 shows the measurements in the strain sweep. The R dough presented the highest linear viscoelastic (LVE) limit (oscillation strain ~0.6%) compared to SCD24_C (oscillation strain ~0.3%) (Figure 5(a)).

The *G*′ modulus of the R sample drop was caused by the application of this strain oscillation, which indicates that the structural rupture of the dough occurs beyond this level of deformation. The data showed that the storage modulus (*G*′) is greater than the loss modulus (*G*^″^) at a lower oscillatory stress, indicating that the samples are in a solid or elastic regime and can store more energy, which they lose as heat when they are deformed by an increasing oscillatory strain. The SDC24_C sample exhibits the convergence of *G*′ and *G*^″^ at a lower strain value than sample R, and a high oscillatory strain value demonstrates interchanged moduli, *G*^″^ > *G*′, demonstrating that the material is dissipating more energy in the form of heat than is stored elastically. This behavior indicates that the dynamic functions *G*′ and *G*^″^ of the samples are independent of deformation in the LVE region.

Figure 5(b) shows the texture map and the classification of materials in four quadrants using the stress rates with the oscillation strain at the limit of the LVE region and the crossover point (point of flow) (*G*′ = *G*^″^). The sample R in quadrant 2 (lower right) is closer to the “rubbery” region, while SCD24_C in quadrant 3 (top right) is closer to the “tough” region with some tendency to the “brittle” region.

Regarding the strain sweep measurements, the two doughs exhibited a low-loss modulus (*G*^″^), indicating that they behave as an elastic-solid material [51] and are less prone to deformation, evidencing a robust interatomic bond, except when there is high oscillatory stress, where they are more likely to flow under pressure, as evidenced in the SCD24_C samples. This characteristic is the typical behavior of soft gels that show a dominant elastic behavior at high frequencies and a dominant viscous response at low frequencies, as reported by Sadat and Joye [52]. Likewise, greater magnitudes in the *G*′ and *G*^″^ moduli in SCD24_C, with respect to R, could be related to protein denaturation and the partial gelatinization of starch granules, producing a loss of crystallinity and the irreversible swelling of starch granules [53, 54], which could be generated by the effect of the pretreatment for SCD24_C that was carried out with dry heat for 24 h.

The SCD24_C dough exhibited a higher LVE limit and showed a higher G. The results indicate that the dough is more resistant and stable, which could be attributed to the fact that it forms a network structure with stronger microstructural interactions compared to R, which could form a weaker and more brittle network in the range of deformation studied [55] according to the texture maps' results, which is consistent with Azeem et al. [56], who state that the particle size can affect the textural properties.

#### 3.6.3. Frequency Sweep Test

**Figure 6:**
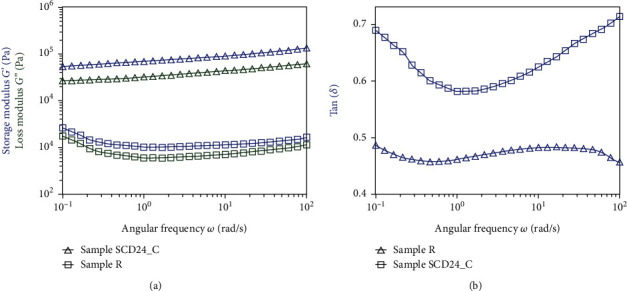
Frequency sweep of bean samples SCD24_C and raw. (a) Response of storage modulus (*G*′ (blue)) and loss modulus (*G*^″^ (green)) versus the angular frequency; (b) tan(*δ*) = *G*^″^/*G*′ versus the angular frequency of the samples at 25°C.

Figure 6 shows the frequency sweep measurements, the response of *G*′, *G*^″^ (Figure 6(a)), and tan(*δ*) = *G*^″^/*G*′ (Figure 6(b)) versus the angular frequency of the doughs of R and SCD24_C at 25°C. The two samples showed *G*′ > *G*^″^ magnitudes with no observable crossing and a tan(*δ*) < 1. For sample R, the values of *G*′ and *G*^″^ showed a slight decrease at low frequencies (0.01–1 rad/s) and then a slight increase at higher frequencies (1–100 rad/s), showing that they are relatively independent of frequency. This suggests a reduction of molecular rearrangement and the formation of strong gels. For sample SDC24_C, the *G*′ and *G*^″^ moduli presented a dependency with increasing frequency. With respect to R, the SDC24_C dough presented higher values of *G*′ and *G*^″^, as shown in Figure 6(a), indicating that the pretreatment increased the system's solid behavior. Likewise, dough SCD24_C, with a larger particle size (242 *μ*m), compared to R, with a smaller particle size (179.1 *μ*m), showed lower tan (*δ*) values (Figure 6(b)) and a more rigid texture.

In the frequency sweep, the two samples studied showed that the modulus of elasticity (*G*′) was greater than the viscous modulus (*G*^″^), no observable crossover between *G*′ and *G*^″^, and the tan (*δ*) was <1, which suggests that the elastic component is dominant, a typical structure of soft gels with cross-linked polymers, since the network prevents the material from flowing, showing a solid-elastic behavior at the frequencies studied [52]. Likewise, the values of *G*′ and *G*^″^ increased constantly with frequency (Figure 6), which could indicate an increase in molecular interactions and a strengthening of the microstructure, showing relaxation processes where the dispersions can be characterized as weak gels [45]. This behavior was consistent with the research of Lin and Fernández-Fraguas [13], who reported a similar behavior in heat-treated bean flours. The upper rates of *G*′ and *G*′′ of SCD24_C reflect the formation of a more rigid structure compared to the untreated samples R, indicating molecular interactions and structural modifications caused by heat treatment, and that could form a high-density three-dimensional network of cross-linked proteins and starch when gelatinization occurs. Probably, the macroscopic expression was the consequence of microlevel changes of starch and protein [57].

#### 3.6.4. Temperature Ramp

**Figure 7:**
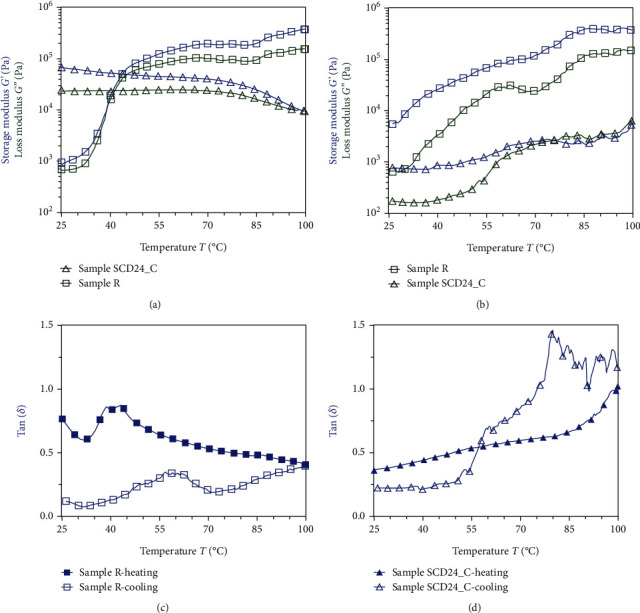
Dynamic viscoelasticity of SCD24_C and raw doughs during the (a) heating and (b) cooling cycle (25°C-95°C-25°C) at a constant strain of 2% and frequency of 1 Hz. Values of tan (*δ*): (c) for raw and (d) SCD24_C.

Figure 7 shows the dynamic viscoelasticity of the bean doughs during the heating (Figure 7(a)) and cooling (Figure 7(b)) cycle (25°C–95°C–25°C) at a constant strain and frequency of 2% and 1 Hz (6.28 rad/s), respectively. For R, the *G*′ and *G*′′ estimates gradually increased with increasing temperature and showed a significant increase in *G*′ around 35°C. The R sample presented a peak in tan (*δ*) values between 40°C and 45°C (Figure 7(c)). On the other hand, for SCD24_C, the moduli (*G*′ and *G*′′) decreased slowly with the increase in temperature, and tan (*δ*) gradually increased with increasing temperature (Figure 7(d)).

The control (R) exhibited the highest amount of the modulus *G*′ around 35°C, which is consistent with that observed in bean flours by Romero and Zhang [58], who raised that the increase of the modulus *G*′ is due to the swelling of the starch granules [59]. On the other hand, for SCD24_C, the *G*′ and *G*^″^ moduli tended to decrease, presenting less rigidity with the increase in temperature, which indicates that the material went into a viscous state. However, the treatment presented an increase in tan (*δ*) value at higher temperatures, which may indicate a change towards a more viscous behavior and a decrease in the material's ability to resist deformation when the material is subjected to heating.

The rheological behavior can be assessed by examining the degree of gelatinized starch and its cross-linking with proteins. The SCD24 treatment was characterized by a weak gel associated with a lesser leaching of amylose. Moreover, the breaking of crystalline granules after thermal treatment could lead to the formation of swelling granules of amylopectin with increased flexibility, thereby affecting starch digestion through microstructural modifications. Additionally, protein denaturation could promote protein aggregation and phase separation, which would impact gel weakening. In the temperature ramp test, the SCD24_C treatment exhibited the lowest temperature for *G*′, indicating a lower crystalline structure (structural integrity) compared to R, and suggesting easier disintegration, which correlates with higher digestibility and estimated glycemic index (eGI).

On the other hand, the technological and functional properties and rheological behavior of bean flour indicate its potential for products with high water absorption, which can result in foods that are soft and viscous, suitable for use in doughs, custards, emulsified meat products, soups, or drink mixtures. Additionally, its high amylose content makes it suitable for fried products with low oil absorption capacity.

## 4. Conclusions

The type of pretreatment used to convert beans into flour could modify its technological properties. Drying at 120°C per 3 h and drying at 75°C per 24 h influenced the WAI increase and WSI decrease. Moreover, the rate of starch hydrolysis increased, thus influencing the rise in eGI. However, the flours obtained from the pretreatment soaking, cooking, and dehydration processes required to reduce antinutritional compounds and improve sensory aspects allowed classifying this type of flour within low-glycemic and similar foods that are also good sources of protein and resistant starch. Additionally, the bean flours obtained with the three pretreatments presented phenolic compounds with bioactive potentials and health-promoting properties, such as catechin, epicatechin, ferulic acid, and quercetin. Thus, promoting their incorporation into food design could increase their intake and significantly improve the regulation of the postprandial glycemic index. Likewise, particle sizes influenced the technological characterization of flours and the postprandial glycemic index. Consequently, the fractionation of flour into larger particles is an industrially viable alternative to obtaining low-glycemic index flour.

The study of the rheological behavior revealed properties of the SCD24_C and R flours, finding that the doughs are more fluid at a higher rate of deformation, which is characteristic of a pseudoplastic. The pretreatment of the bean influenced the starch structure and the interaction of its components, contributing to the increase of its water absorption capacity and the viscosity of the dough, resulting in a more resistant and stable material compared to the raw material R. The doughs of the SCD24_C flour behaved as gel-viscous weak at low frequencies and gel-elastic dominant at high frequencies.

## Figures and Tables

**Table 1:** Starch fractions, rapidly digested starch (RDS), slowly digested starch (SDS), resistant starch (RS), and amylose contents of bean flour.

	Treatment	Fraction	RDS	SDS	RS	Amylose %
Bean	R	Whole	6.14 ± 0.45^ab^	2.95 ± 0.3^a^	28.57 ± 0.75^de^	67.93 ± 13.71^c^
	D	Whole	8.30 ± 0.65^b^	5.28 ± 0.9^c^	24.09 ± 1.18^b^	57.61 ± 7.39^abc^
	SCD3	Whole	7.53 ± 0.08^c^	6.80 ± 0.29^d^	23.34 ± 0.3^b^	45.10 ± 4.64^ab^
		Fine	16.35 ± 0.78^d^	2.78 ± 0.53^bc^	18.54 ± 1.31^a^	43.34 ± 2.58^a^
		Coarse	7.64 ± 1.94^b^	4.96 ± 0.12^c^	25.07 ± 1.82^cd^	42.95 ± 4.98^a^
	SCD24	Whole	13.91 ± 1.58^cd^	1.18 ± 0.17^a^	22.58 ± 1.4^bc^	65.51 ± 2.58^bc^
		Fine	14.62 ± 1.75^cd^	3.95 ± 0.43^bc^	19.10 ± 2.18^b^	41.37 ± 1.93^a^
		Coarse	3.68 ± 0.99^a^	3.43 ± 0.63^b^	30.55 ± 1.63^e^	54.97 ± 9.9^abc^

Values are means ± SEM of three replicates. Different letters (a–e) show statistically significant differences *P* < 0.05 by Tukey.

**Table 2:** Composition of flavonoids and phenolic acids in the bean flours.

Pretreatment	Catechin (mg kg^−1^)	Epicatechin (mg kg^−1^)	Ferulic acid (mg kg^−1^)	Quercetin (mg kg^−1^)	Quercetin-3-glucoside (mg kg^−1^)	Pelargonidin-3-glucoside (mg kg^−1^)
R	23.7 ± 8.6^ab^	1.55 ± 0.5^a^	11.45 ± 0.5^b^	2.75 ± 0.4^bc^	22.15 ± 3.5^b^	14.95 ± 3.7^a^
D	33.9 ± 3.5^b^	2.15 ± 0.1^a^	9.5 ± 1.8^b^	1.65 ± 0.5^a^	25.1 ± 3.4^b^	25 ± 1.8^b^
SCD3_F	46.85 ± 3.7^c^	11 ± 1.6^b^	10.6 ± 0.3^b^	3.8 ± 0.3^d^	8.3 ± 0.1^a^	<1.0
SCD3_C	49.1 ± 4.4^c^	9.35 ± 0.1^b^	11.2 ± 0.8^b^	3.6 ± 0.1^cd^	9.65 ± 0.8^a^	<1.0
SCD24_F	12.45 ± 0.1^a^	1.05 ± 1.5^a^	4.75 ± 0.2^a^	2.15 ± 0.6^ab^	6.45 ± 1.3^a^	<1.0
SCD24_C	14.7 ± 4.8^a^	1.55 ± 0.8^a^	4.65 ± 0.8^a^	2.65 ± 0.4^abc^	6.45 ± 0.2^a^	<1.0

Values are means ± SEM of two replicates. Different letters (a–d) show statistically significant differences *P* < 0.05 by Tukey.

## Data Availability

The data generated or analyzed during this study are available upon reasonable request from the corresponding author.
